# Supplementary material for: Market Imitation and Win-Stay Lose-Shift Strategies Emerge as Unintended Patterns in Market Direction Guesses
Source: PLoS One. 2016 Aug 17;11(8):e0159078. doi: 10.1371/journal.pone.0159078 (PMC4988703; doi:10.1371/journal.pone.0159078)
Supplement: S1 File — In the S1 File (PDF) we present further details about the experiment, statistical tests and additional information. (PDF) [file pone.0159078.s001.pdf]

Supplementary Information: Market Imitation and Win-Stay Lose-Shift strategies emerge as unintended patterns in market direction guesses

Mario Gutiérrez-Roig[1], Carlota Segura[1], Jordi Duch[2], and Josep Perelló[1,*]

[1]Departament de Física Fonamental, Universitat de Barcelona. Martí i Franqués 1, E-08028 Barcelona, Spain
[2]Departament d'Enginyeria Informàtica i Matemàtiques, Universitat Rovira i Virgili, Tarragona, Spain
[*]josep.perello@ub.edu

ABSTRACT

This document accounts for the Supplementary Information of the paper entitled "Market Imitation and Win-Stay Lose-Shift strategies emerge as unintended patterns in market direction guesses" authored by Mario Gutiérrez-Roig, Carlota Segura, Jordi Duch and Josep Perelló. Complementary analysis (aggregation of scenarios, cohort analysis, time evolution, conditional Mutual Information and information loss) and definitions (statistical measures, and Mutual information) that underpin arguments in the main paper are detailed here. Additionally, we include some extra information related with the experiment (survey questions, replication of the experiment and tutorial screenshots).

Statistics

As we are facing a binomial process where only two exclusive states are possible ("up"/"down", "correct"/"wrong" and "repeat"/"change") we calculate the sample probability as

$$p(X) = s_X/n \tag{1}$$

where X is the state , s_X is the number of X events in the sample and n the total number of events in the sample. The associated standard error (that is: the Standard Deviation, SD) to this probability is

$$\mathrm{SD}(X) = \sqrt{\frac{p(X)(1-p(X))}{n}}. \tag{2}$$

Finally, the standard error when calculating the difference between two sample probabilities $p_1(X)$ and $p_2(X)$ reads,

$$\mathrm{SD}_{1-2}(X) = \sqrt{\frac{p_1(X)(1-p_1(X))}{n_1} + \frac{p_2(X)(1-p_2(X)}{n_2}} \tag{3}$$

where n_1 and n_2 account for the number of X events in sample s_1 and s_2 respectively. All these definitions are thus applied in the error analysis along the paper.

Aggregation of Scenarios

The goal of aggregating all scenarios is to present results based on more robust statistics. However, such aggregation can only be justified if the results for every scenario are comparable to the aggregated data and do not show very significant differences. In order to test such thing, we performed the same analysis presented in the paper for each of the scenarios described in the "Materials and Methods" section of the main paper. As we can see in the tables below, conditional probabilities associated to Market Imitation strategy (S1 Table A and S1 Table B), to Win-Stay Lose-Shift strategy (S1 Table C and S1 Table D), and to follow one of the two strategies, are not significantly different from the aggregated case. Additionally, S1 Figure A displays that the probability to follow any of those strategies reproduces the same pattern independently of the scenario.

Cohort	Aggregate	1C	1I	2C	2I	3C	3I
				Time (seconds)			
0-5	0.732 ± 0.006	0.739 ± 0.016	0.730 ± 0.014	0.725 ± 0.012	0.733 ± 0.015	0.732 ± 0.013	0.734 ± 0.013
5-10	0.705 ± 0.009	0.738 ± 0.022	0.715 ± 0.019	0.610 ± 0.036	0.730 ± 0.019	0.680 ± 0.024	0.694 ± 0.025
10-15	0.659 ± 0.017	0.678 ± 0.028	0.625 ± 0.171	0.667 ± 0.073	0.631 ± 0.032	0.670 ± 0.046	0.660 ± 0.047
15-20	0.574 ± 0.031	0.642 ± 0.047	–	0.667 ± 0.122	0.526 ± 0.057	0.346 ± 0.093	0.600 ± 0.078
20-25	0.575 ± 0.047	0.646 ± 0.069	–	0.400 ± 0.219	0.561 ± 0.078	0.667 ± 0.193	0.385 ± 0.135
25-30	0.673 ± 0.065	0.731 ± 0.087	–	–	0.539 ± 0.138	0.500 ± 0.354	0.800 ± 0.127
				Information (consulted panels)			
0	0.733 ± 0.006	0.758 ± 0.018	0.751 ± 0.016	0.708 ± 0.011	0.766 ± 0.017	0.718 ± 0.014	0.743 ± 0.017
1	0.680 ± 0.009	0.686 ± 0.033	0.661 ± 0.024	–	0.622 ± 0.024	0.701 ± 0.017	0.696 ± 0.015
2	0.720 ± 0.017	0.714 ± 0.030	0.726 ± 0.031	–	0.720 ± 0.029	–	–
3	0.731 ± 0.021	0.760 ± 0.034	0.740 ± 0.036	–	0.697 ± 0.036	–	–
4	0.680 ± 0.019	0.650 ± 0.031	0.723 ± 0.037	–	0.685 ± 0.033	–	–
5	0.691 ± 0.038	0.662 ± 0.055	0.790 ± 0.094	–	0.696 ± 0.061	–	–
6	0.717 ± 0.025	0.722 ± 0.034	0.783 ± 0.061	–	0.684 ± 0.043	–	–
				Expert (aggregated vs. consulting expert)			
agg.	0.740 ± 0.005	0.779 ± 0.014	0.761 ± 0.013	0.708 ± 0.011	0.761 ± 0.013	0.715 ± 0.011	0.747 ± 0.012
expert	0.637 ± 0.010	0.650 ± 0.017	0.661 ± 0.019	–	0.627 ± 0.018	0.649 ± 0.045	0.574 ± 0.029

S1 Table A. Probability to choose "up" after market has gone "up" segregating by scenarios. First column shows the values for the three different observables, time, information and expert advice, tested in the Figure 6 of the main paper. Second column denotes the aggregated probability and the Standard Deviation regardless of the scenarios. The rest columns contain such measures segregated by the scenarios described in the "Materials and Methods" section of the main paper.

Value	Aggregate	1C	1I	2C	2I	3C	3I
				Time (seconds)			
0-5	0.539 ± 0.007	0.530 ± 0.021	0.536 ± 0.017	0.553 ± 0.015	0.505 ± 0.019	0.547 ± 0.017	0.551 ± 0.017
5-10	0.524 ± 0.012	0.532 ± 0.028	0.505 ± 0.024	0.592 ± 0.043	0.507 ± 0.027	0.536 ± 0.029	0.518 ± 0.032
10-15	0.525 ± 0.021	0.530 ± 0.035	0.429 ± 0.187	0.625 ± 0.086	0.533 ± 0.039	0.525 ± 0.064	0.463 ± 0.055
15-20	0.533 ± 0.032	0.561 ± 0.050	–	0.539 ± 0.138	0.480 ± 0.058	0.630 ± 0.093	0.485 ± 0.087
20-25	0.370 ± 0.050	0.375 ± 0.077	–	0.333 ± 0.272	0.387 ± 0.088	0.300 ± 0.145	0.375 ± 0.171
25-30	0.400 ± 0.069	0.333 ± 0.096	–	–	0.533 ± 0.129	0.429 ± 0.187	0.250 ± 0.217
				Information (consulted panels)			
0	0.543 ± 0.008	0.542 ± 0.025	0.516 ± 0.021	0.558 ± 0.014	0.506 ± 0.024	0.543 ± 0.018	0.568 ± 0.022
1	0.510 ± 0.011	0.483 ± 0.038	0.486 ± 0.030	–	0.484 ± 0.029	0.543 ± 0.021	0.513 ± 0.018
2	0.496 ± 0.023	0.455 ± 0.038	0.556 ± 0.040	–	0.484 ± 0.040	–	–
3	0.530 ± 0.024	0.510 ± 0.040	0.596 ± 0.041	–	0.479 ± 0.045	–	–
4	0.559 ± 0.023	0.579 ± 0.034	0.556 ± 0.052	–	0.536 ± 0.037	–	–
5	0.556 ± 0.048	0.546 ± 0.067	0.400 ± 0.155	–	0.605 ± 0.075	–	–
6	0.537 ± 0.033	0.546 ± 0.048	0.516 ± 0.090	–	0.532 ± 0.052	–	–
				Expert (aggregated vs. consulting expert)			
agg.	0.538 ± 0.007	0.536 ± 0.020	0.528 ± 0.017	0.558 ± 0.014	0.498 ± 0.019	0.541 ± 0.014	0.546 ± 0.016
expert	0.515 ± 0.011	0.513 ± 0.020	0.519 ± 0.024	–	0.513 ± 0.020	0.567 ± 0.049	0.494 ± 0.031

S1 Table B. Probability to choose "down" after market has gone "down" segregating by scenarios. First column shows the values for the three different observables, time, information and expert advice, being presented in Figure 6 of the main paper. Second column denotes the aggregated probability and the Standard Deviation regardless of the scenarios. The rest columns contain such measures segregated by the scenarios described in the "Materials and Methods" section of the main paper.

Value	Aggregate	1C	1I	2C	2I	3C	3I
Time (seconds)							
0-5	0.697 ± 0.006	0.674 ± 0.017	0.688 ± 0.015	0.698 ± 0.013	0.697 ± 0.016	0.731 ± 0.014	0.685 ± 0.015
5-10	0.683 ± 0.010	0.722 ± 0.022	0.674 ± 0.020	0.643 ± 0.034	0.714 ± 0.020	0.652 ± 0.025	0.659 ± 0.026
10-15	0.640 ± 0.017	0.635 ± 0.029	0.556 ± 0.166	0.717 ± 0.066	0.617 ± 0.032	0.654 ± 0.047	0.663 ± 0.047
15-20	0.555 ± 0.030	0.583 ± 0.046	–	0.600 ± 0.126	0.512 ± 0.055	0.556 ± 0.096	0.548 ± 0.077
20-25	0.574 ± 0.046	0.625 ± 0.070	–	0.250 ± 0.217	0.565 ± 0.073	0.625 ± 0.171	0.444 ± 0.166
25-30	0.607 ± 0.063	0.586 ± 0.091	–	–	0.647 ± 0.116	0.500 ± 0.204	0.667 ± 0.157
Information (consulted panels)							
0	0.702 ± 0.007	0.691 ± 0.021	0.693 ± 0.018	0.689 ± 0.012	0.743 ± 0.019	0.717 ± 0.016	0.698 ± 0.019
1	0.650 ± 0.009	0.619 ± 0.033	0.631 ± 0.025	–	0.604 ± 0.024	0.686 ± 0.017	0.657 ± 0.015
2	0.680 ± 0.019	0.696 ± 0.032	0.683 ± 0.033	–	0.664 ± 0.031	–	–
3	0.695 ± 0.021	0.692 ± 0.035	0.735 ± 0.036	–	0.660 ± 0.038	–	–
4	0.678 ± 0.019	0.650 ± 0.030	0.726 ± 0.037	–	0.679 ± 0.031	–	–
5	0.629 ± 0.038	0.631 ± 0.053	0.667 ± 0.103	–	0.611 ± 0.066	–	–
6	0.682 ± 0.025	0.665 ± 0.036	0.638 ± 0.070	–	0.724 ± 0.040	–	–
Expert (aggregated vs. consulting expert)							
agg.	0.711 ± 0.006	0.729 ± 0.016	0.709 ± 0.014	0.689 ± 0.012	0.728 ± 0.015	0.708 ± 0.012	0.716 ± 0.013
expert	0.610 ± 0.009	0.613 ± 0.017	0.637 ± 0.020	–	0.621 ± 0.017	0.640 ± 0.043	0.520 ± 0.027

S1 Table C. Probability to "repeat" the previous decision after a "success" segregating by scenarios. First column shows the values for the three different observables, time, information and expert advice, tested in the Figure 6 of the main paper. Second column denotes the aggregated probability and the Standard Deviation regardless of the scenarios. The rest columns contain such measures segregated by the scenarios described in the "Materials and Methods" section of the main paper.

Value	Aggregate	1C	1I	2C	2I	3C	3I
Time (seconds)							
0-5	0.596 ± 0.007	0.620 ± 0.019	0.598 ± 0.016	0.599 ± 0.014	0.568 ± 0.018	0.575 ± 0.015	0.619 ± 0.016
5-10	0.555 ± 0.012	0.550 ± 0.028	0.559 ± 0.023	0.531 ± 0.047	0.544 ± 0.026	0.567 ± 0.029	0.570 ± 0.031
10-15	0.546 ± 0.022	0.584 ± 0.036	0.500 ± 0.204	0.536 ± 0.094	0.546 ± 0.040	0.550 ± 0.064	0.464 ± 0.054
15-20	0.553 ± 0.033	0.629 ± 0.051	–	0.615 ± 0.135	0.493 ± 0.060	0.423 ± 0.097	0.548 ± 0.089
20-25	0.367 ± 0.051	0.400 ± 0.077	–	0.500 ± 0.250	0.346 ± 0.093	0.250 ± 0.153	0.333 ± 0.136
25-30	0.439 ± 0.078	0.476 ± 0.109	–	0.000 ± 0.000	0.364 ± 0.145	0.333 ± 0.272	0.600 ± 0.219
Information (consulted panels)							
0	0.597 ± 0.007	0.642 ± 0.023	0.597 ± 0.019	0.591 ± 0.013	0.577 ± 0.022	0.571 ± 0.017	0.636 ± 0.020
1	0.549 ± 0.011	0.557 ± 0.040	0.531 ± 0.029	–	0.500 ± 0.030	0.561 ± 0.021	0.563 ± 0.018
2	0.562 ± 0.022	0.507 ± 0.035	0.613 ± 0.039	–	0.580 ± 0.037	–	–
3	0.567 ± 0.025	0.561 ± 0.042	0.600 ± 0.041	–	0.535 ± 0.044	–	–
4	0.551 ± 0.024	0.571 ± 0.036	0.554 ± 0.052	–	0.525 ± 0.040	–	–
5	0.643 ± 0.048	0.578 ± 0.074	0.625 ± 0.171	–	0.711 ± 0.068	–	–
6	0.585 ± 0.033	0.640 ± 0.046	0.733 ± 0.081	–	0.466 ± 0.053	–	–
Expert (aggregated vs. consulting expert)							
agg.	0.590 ± 0.006	0.620 ± 0.018	0.598 ± 0.016	0.591 ± 0.013	0.577 ± 0.017	0.567 ± 0.014	0.602 ± 0.015
expert	0.542 ± 0.011	0.551 ± 0.021	0.557 ± 0.024	–	0.510 ± 0.021	0.570 ± 0.051	0.561 ± 0.033

S1 Table D. Probability to "change" the previous decision that resulted "wrong" guessing segregating by scenarios. First column shows the values for the three different observables, time, information and expert advice, tested in the Figure 6 of the main paper. Second column denotes the aggregated probability and the Standard Deviation regardless of the scenarios. The rest columns contain such measures segregated by the scenarios described in the "Materials and Methods" section of the main paper.

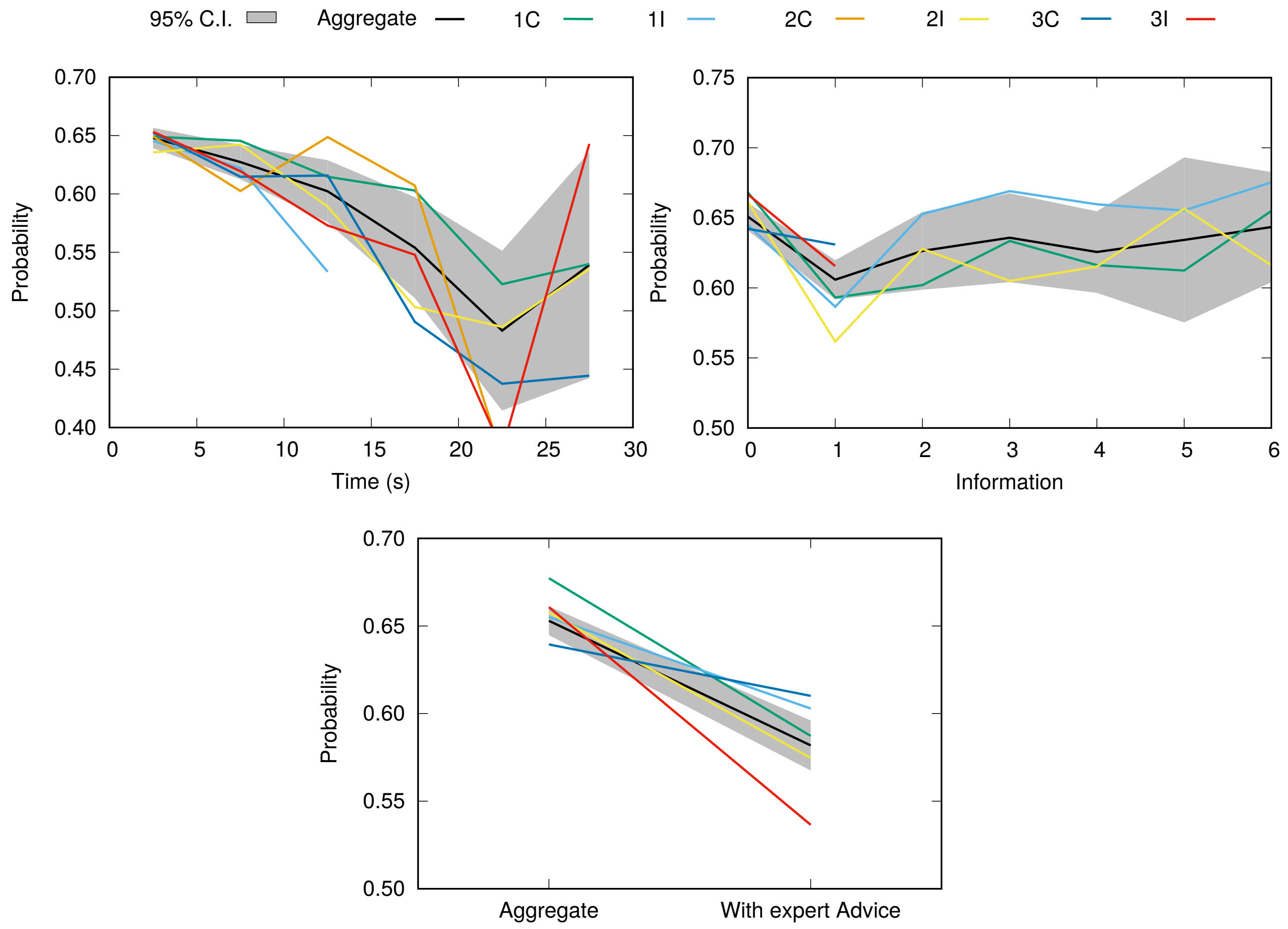

S1 Figure A. Probability to "Follow" either Market Imitation and Win-Stay Lose-Shift strategy depending on time, information and expert, and segregated by scenarios. The three plots are equivalent to the Figure 6 of the main paper. Top-left plot shows the probability depending on the time spent before making a decision, top-right subfigure plots the probability depending on the number of panels consulted, and bottom plot accounts for the probability depending on having consulted the expert advice. Color lines represent the values for different scenarios while black line represents the aggregate values. Shaded area corresponds to the 95% Confidence Interval.

	p(Up-Up)	p(Down-Down)	p(Sucess-Repeat)	p(Fail-Change)	p(Follow)
With Scenario 4	0.712 ± 0.004	0.514 ± 0.005	0.680 ± 0.004	0.571 ± 0.005	0.630 ± 0.003
Without Scenario 4	0.717 ± 0.005	0.524 ± 0.006	0.690 ± 0.005	0.570 ± 0.006	0.635 ± 0.004

S1 Table E. Conditional Probabilities compared with and without Fourth Scenario. First column refers to the two cases. Second and third columns to the probability to choose "up" after the market has raised and to the probability to choose "down" after a market drop respectively. Fifth and fourth column, account for the probability to repeat a right decision and for the probability to change the previous decision that resulted wrong. The last column is the probability to follow any of Market Imitation or Win-Stay Lose-Shfit strategy as described in the main paper

Cohort Analysis

Here we present some plots and tables to support the aggregation of data by age, gender and educational studies, and we only find significant bias in some concrete cases. S1 Table F shows how the bias towards choosing "up" is totally general and fully described by the aggregated probabilities of $p(\uparrow) = 0.6060$ and $p(\downarrow) = 0.3940$. All groups except the elderly (more than 65 years old) and the one that do not specify the educational level can be described by these two probabilities. Indeed, these two groups that significantly deviate from the aggregated probability values are very small, representing 0.81% and 1,08% of the population sample, respectively.

Cohort Group	Decisions		"up"			"down"		
Male	11,845	0.6425	7,124	0.6014	-0.79 SD units	4,721	0.3986	0.79 SD units
Female	6,591	0.3575	4,049	0.6143	1.19 SD units	2,542	0.3857	-1.19 SD units
0-15 y.o.	5,462	0.2963	3,260	0.5969	-1.21 SD units	2,202	0.4031	1.21 SD units
16-25 y.o.	2,316	0.1256	1,393	0.6015	-0.42 SD units	923	0.3985	0.42 SD units
26-35 y.o.	4,999	0.2712	3,023	0.6047	-0.16 SD units	1,976	0.3953	0.16 SD units
36-45 y.o.	3,311	0.1796	4,049	0.6143	0.48 SD units	1,290	0.3896	-0.48 SD units
46-55 y.o.	1,726	0.0936	1,086	0.6292	1.91 SD units	640	0.3708	-1.91 SD units
56-65 y.o.	473	0.0257	286	0.6047	-0.06 SD units	187	0.3953	0.06 SD units
66+ y.o.	149	0.0081	104	0.6980	**2.43 SD units**	45	0.3020	**-2.43 SD units**
None	324	0.0176	191	0.5895	-0.60 SD units	133	0.4105	0.60 SD units
Primary	3,442	0.1869	2,065	0.5999	-0.67 SD units	1,377	0.4001	0.67 SD units
Secondary	2,519	0.1366	1,531	0.6078	0.17 SD units	988	0.3922	-0.17 SD units
High School	2,191	0.1188	1,331	0.6075	0.13 SD units	860	0.3925	-0.13 SD units
University	9,761	0.5295	5,963	0.6099	0.63 SD units	3,808	0.3901	-0.63 SD units
Unavailable	199	0.0108	102	0.5126	**-2.62 SD units**	97	0.4874	**2.62 SD units**

S1 Table F. Cohort analysis in "up"/"down" decisions. First column indicates the cohort, which belongs to one of the three blocks: Gender, Age and Education Level. Second and third columns contain the number of actions from each group and its population ratio. Fourth, fifth and sixth columns show the number of times each group member selected "up", its fraction over the total number of events and the difference with the aggregated probability $p(\uparrow)$ in Standard Deviation (SD) units calculated using Equation (3). The same is done in seventh, eighth and ninth columns for the "down" choices. We highlight the discrepancies in bold for those cases where the probability deviates beyond 1.96 SD units threshold, that corresponds to 95% confidence interval assuming Bernouilli trials.

On the other hand, we can study the cohort effect in the probabilities of repeating decisions (see S1 Table G). The aggregated probability to repeat any decision is 0.5612 in front of 0.4388 chances of changing the decision previously made. In contrast of the cohort analysis for decision direction, here we find a very significant deviation between age groups. The most numerous, the youngest, have 0.4906 to repeat a decision, almost 9 SD units below reference level and very close to meaningful $1/2$. Conversely, the other groups compensate this strong deviation by significantly being over the reference level, concretely in values around 0.60. Besides, this age discrimination has also a strong influence when looking at educational level groups. "None", "Primary" and "Secondary" education corresponds precisely to 0-15 age range in Spanish education system. This explains the low values ranging from 0.48 to 0.53 in this three groups while is compensated in higher educational levels with probabilities around 0.60.

Finally, we also analyse the probability to follow any of the two strategies described in the main paper (*Market Imitation* and *Win-Stay Lose-Shift* emerging strategies) by discriminating groups in the three different cohorts (S1 Table H). In contrast with the cohort analysis above, here we find many groups that are not well explained by the aggregate probability to follow any of strategies which equals to 0.6344 ± 0.0036. Some of them are not relevant due to the small number of events, like "56-65 years old" group in age cohort block or "None" and "Unavailable" groups in educational cohort block. Despite we find two groups in the age cohort block that deviate from aggregated probability ("26-35 years old" and "46-55 years old"), is not possible to establish any trend that indicates a sort of dependence in age when fulfilling the strategies. Quite the same occurs in the educational cohort block. Although one could be tempted to hypothesize that more education implies less probability to follow these intuitive strategies looking at the first four groups, "University" group (the most numerous) should confirm that trend and clearly does not. However, in gender cohort block we find a notorious deviation from the aggregate probability: Females are significantly more prone to follow the strategies than males. There is 0.0529 points of difference between them, which equals to 8.13 SD units.

In summary, is quite clear that all cohort groups present a probability to follow the intuitive strategies that oscillates between

Cohort Group	Decisions		"repeat"			"change"		
Male	11,324	0.6426	6,410	0.5661	0.81 SD units	4,914	0.4339	-0.81 SD units
Female	6,297	0.3574	3,479	0.5525	-1.19 SD units	2,818	0.4475	1.19 SD units
0-15 y.o.	5,230	0.2968	2,566	0.4906	**-8.98 SD units**	2,664	0.5094	**8.98 SD units**
16-25 y.o.	2,215	0.1257	1,268	0.5725	1.01 SD units	947	0.4275	-1.01 SD units
26-35 y.o.	4,775	0.2710	2,835	0.5937	**4.05 SD units**	1,940	0.4063	**-4.05 SD units**
36-45 y.o.	3,167	0.1797	1,878	0.5930	**3.35 SD units**	1,289	0.4070	**-3.35 SD units**
46-55 y.o.	1,639	0.0930	1,003	0.6120	**4.03 SD units**	636	0.3880	**-4.03 SD units**
56-65 y.o.	452	0.0257	251	0.5553	-0.25 SD units	201	0.4447	0.25 SD units
66+ y.o.	143	0.0081	88	0.6154	1.33 SD units	55	0.3846	-1.33 SD units
None	310	0.0176	160	0.5161	-1.57 SD units	150	0.4839	1.57 SD units
Primary	3,296	0.1870	1,593	0.4833	**-8.22 SD units**	1,703	0.5167	**8.22 SD units**
Secondary	2,412	0.1369	1,288	0.5340	**-2.51 SD units**	1,124	0.4660	**2.51 SD units**
High school	2,094	0.1188	1,230	0.5874	**2.30 SD units**	864	0.4126	**-2.30 SD units**
University	9,318	0.5288	5,553	0.5959	**5.51 SD units**	3,765	0.4041	**-5.51 SD units**
Unavailable	191	0.0108	65	0.3403	**-6.40 SD units**	126	0.6597	**6.40 SD units**

S1 Table G. Cohort analysis in "repeat"/"change" decisions. The table summarizes the cohort analysis results for the different groups and in relation whether they repeat consecutive decisions. First column indicates the cohort, which belongs to one of the three blocks: Gender, Age and Education level. Second and third column contain the number of total actions from each group and its population ratio. Fourth, fifth and sixth columns show the number of times each group repeated decisions, its corresponding fraction and the difference with the aggregated probability to repeat a decision in Standard Deviation (SD) units calculated using Equation (3). The same is done in seventh, eighth and ninth columns that account for those actions which have changed with respect the decision taken in the previous step. The discrepancies in bold account for those cases where the probability deviates beyond 1.96 SD units threshold, that corresponds to 95% confidence interval assuming Bernoulli trials.

Cohort Group	Decisions		"Follow the Strategy"			"Not Follow the Strategy"		
Male	11,324	0.6426	6,970	0.6155	**-3.24 SD units**	4,354	0.3845	**3.24 SD units**
Female	6,297	0.3574	4,209	0.6684	**4.89 SD units**	2,088	0.3316	**-4.89 SD units**
0-15 y.o.	5,230	0.2968	3,386	0.6474	1.73 SD units	1,844	0.3526	-1.73 SD units
16-25 y.o.	2,215	0.1257	1,389	0.6271	-0.67 SD units	826	0.3729	0.67 SD units
26-35 y.o.	4,775	0.2710	2,909	0.6092	**-3.17 SD units**	1,866	0.3908	**3.17 SD units**
36-45 y.o.	3,167	0.1797	1,998	0.6309	-0.38 SD units	1,169	0.3691	0.38 SD units
46-55 y.o.	1,639	0.0930	1,097	0.6693	**2.87 SD units**	542	0.3307	**-2.87 SD units**
56-65 y.o.	452	0.0257	313	0.6925	**2.64 SD units**	139	0.3075	**-2.64 SD units**
66+ y.o.	143	0.0081	87	0.6084	-0.63 SD units	56	0.3916	0.63 SD units
None	310	0.0177	213	0.6871	**1.98 SD units**	97	0.3129	**-1.98 SD units**
Primary	3,296	0.1877	2,200	0.6675	**3.69 SD units**	1,096	0.3325	**-3.69 SD units**
Secondary	2,412	0.1373	1,481	0.6140	-1.93 SD units	931	0.3860	1.93 SD units
High school	2,094	0.1192	1,254	0.5989	**-3.14 SD units**	840	0.4011	**3.14 SD units**
University	9,258	0.5272	5,927	0.6402	0.94 SD units	3,331	0.3598	-0.94 SD units
Unavailable	191	0.0109	104	0.5445	**-2.48 SD units**	87	0.4555	**2.48 SD units**

S1 Table H. Cohort Analysis in "Follow the Strategy" and "Not Follow the Strategy". This table summarizes the cohort analysis results in the case whether the different groups are following or not any of the two strategies described at main paper (*Market Imitation* and *Win-Stay Lose-Shift* emerging strategies). First column indicates the cohort, which belongs to one of the three blocks: Gender, Age and Education Level. Second and third column contain the number of total events from each group and its population ratio. Fourth, fifth and sixth columns show the number of times each group followed any of the two strategies, its fraction over the total events and the difference with the aggregated probability to follow the strategy in Standard Deviation (SD) units calculated using Equation (3). The same is done in seventh, eighth and ninth columns for the "Not Follow the Strategy" events. The discrepancies in bold account for those cases where the probability deviates beyond 1.96 SD units threshold, that corresponds to 95% confidence interval assuming Bernoulli trials.

Cohort Group	Time			Information			Expert		
Male	5.399	0.039	**3.91 SD units**	2.127	0.012	**2.71 SD units**	16.58	0.34	1.40 SD units
Female	4.813	0.034	**-7.44 SD units**	2.004	0.011	**-4.99 SD units**	14.87	0.44	**-2.14 SD units**
0-15	3.293	0.027	**-41.27 SD units**	1.626	0.008	**-33.04 SD units**	9.72	0.40	**-12.96 SD units**
16-25	6.801	0.041	**28.97 SD units**	2.814	0.014	**39.91 SD units**	22.97	0.84	**7.67 SD units**
26-35	5.919	0.038	**13.74 SD units**	2.293	0.012	**12.51 SD units**	16.46	0.52	0.83 SD units
36-45	5.831	0.040	**11.78 SD units**	2.073	0.011	-0.63 SD units	17.56	0.66	**2.23 SD units**
46-55	5.466	0.039	**5.13 SD units**	1.876	0.010	**-13.74 SD units**	19.54	0.95	**3.62 SD units**
56-65	6.070	0.036	**17.06 SD units**	2.400	0.013	**18.04 SD units**	29.05	2.08	**6.23 SD units**
+66	4.718	0.027	**-10.23 SD units**	2.053	0.009	**-2.04 SD units**	0.67	0.66	**-21.30 SD units**
None	5.311	0.029	**2.58 SD units**	2.517	0.015	**22.95 SD units**	12.92	1.86	-1.62 SD units
Primary	3.159	0.027	**-44.41 SD units**	1.531	0.008	**-40.07 SD units**	8.03	0.46	**-14.83 SD units**
Secondary	3.876	0.032	**-26.84 SD units**	1.819	0.009	**-18.31 SD units**	13.47	0.68	**-3.42 SD units**
High School	5.537	0.037	**6.59 SD units**	2.056	0.011	-1.70 SD units	18.32	0.82	**2.71 SD units**
University	6.142	0.040	**17.57 SD units**	2.326	0.013	**14.38 SD units**	18.93	0.40	**6.19 SD units**
Unavailable	5.960	0.039	**14.39 SD units**	2.555	0.016	**24.08 SD units**	18.00	20.72	0.74 SD units

S1 Table I. Cohort in the observables: Time, Information and Expert. Here we show the cohort analysis performed for the three studied observables: time spent during making-decision process, amount of information consulted and expert's advice. For each category we plot the mean in the first column, the Standard Deviation (SD) of the mean in the second and the difference with respect to the global mean in Standard Deviation units. The global average values are 5.189 ± 0.37 seconds for time category, 2.083 ± 0.011 for amount of information and $15.07\% \pm 0.27\%$ for expert advice. The discrepancies in bold account for those cases where the probability deviates beyond 1.96 SD units threshold, that corresponds to 95% confidence interval assuming Bernoulli trials.

60% and 70%, values far beyond from a 50% expectable if the decisions were random. Moreover, in every group the probability to follow the strategy is always above its corresponding probability of direction bias and the probability to repeat a decision. Therefore, the strategies are generally followed, no matter which cohort group we look at, but it is also true that in some groups the strategies are followed with more intensity.

Time Evolution along the Experiment

An important feature to look into in this type of experiments is the learning curve. Despite participants played few rounds within the tutorial, a double-check should be done in order to see if the first rounds are statistically different. In the Figure 1b of the main paper we observe how time distribution barely changes along time. S1 Figure B shows how either success probability and the average of information panels consulted remains stable in all rounds. Regarding decision bias "up"/"down" and success ratio, we observe very much the same in all rounds (see S1 Figure C). Moreover, the probability to trust the expert when consulted is also statistically the same when we compare it as a function of how many times the expert has been consulted before (see S1 Figure D). Therefore, we find that all observables are not dependent on the evolution of the game and remain very stable. Based on the fact that there is an absence of learning effects, we do not discard any round in our analysis.

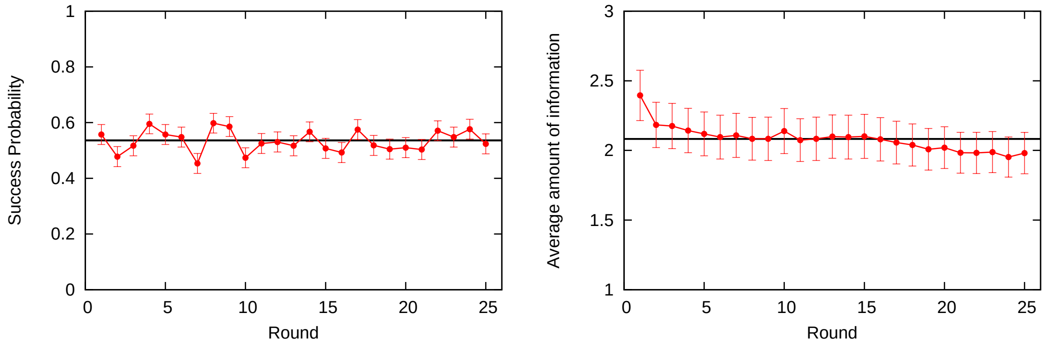

S1 Figure B. **Success and information evolution.** (Left) Aggregated success probability in each round. The error bars represent 1.96 Standard Deviation (SD) units (that is, the 95% confidence interval). The solid black lines denotes the total success probability 0.5359. (Right) Average number of consulted information panels as function of round number. The error bars represent 1.96 SD units (that is, the 95% confidence interval).

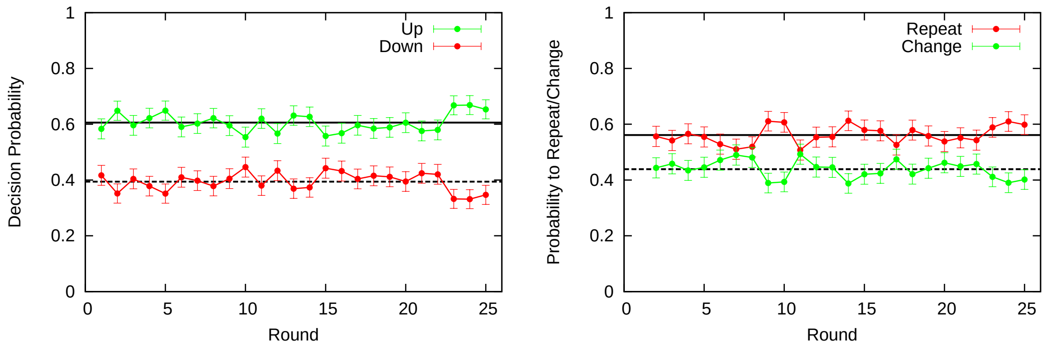

S1 Figure C. **The "up"/"down" and "repeat"/"change" probabilities evolution.** (Left) Aggregated probability to choose "up" (green) or "down" (red) for each round. The error bars represent 1.96 SD units (that is, the 95% confidence interval). The solid black line denotes the aggregated probability to choose "up" placed at 0.6060 and the dashed black line the aggregated probability to choose "down" at 0.3940. (Right) Aggregated probability to choose "up" (green) or "down" (red) for each round. The error bars represent 1.96 SD units (that is, the 95% confidence interval). The solid black line denotes the aggregated probability to repeat the decision (at 0.5612) and the dashed black line the aggregated probability to change the decision (at 0.4388).

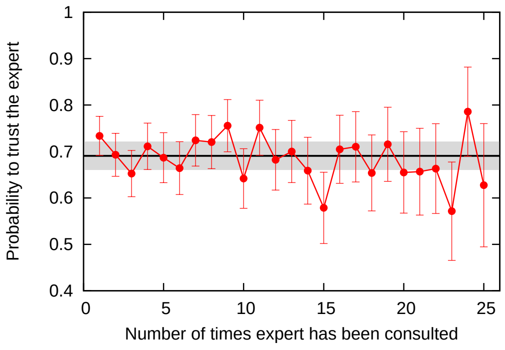

S1 Figure D. **Expert trustiness evolution as a function of the number of times been previously consulted.** The probability to follow the expert advice includes the actual one. Solid black line is the aggregated probability to trust the expert (0.6905). The error bars as well as grey area represent 1.96 SD units (that is, a 95% confidence interval).

Aggregating Strategies

We now demonstrate how the coarse-grained approach reduced by the "Follow the Strategy" and "Not Follow the Strategy" events lead to exactly the same results for both *Market Imitation* and *Win-Stay Lose-Shift* by construction. Firstly, we must note that both strategies need of the previous step in order to take a decision (i.e. they need an stimulus and then respond to that stimulus). Thus, the first decision ("up"/"down") of the game is not selected by using any of the two emerging strategies studied here, since there is no previous stimulus. In second place, either the options ("up"/"down") and the result ("correct"/"wrong") are two mutually exclusive events. Consider, for example, a case where in a specific round the participant decides to choose "up" but the market goes "down". The outcome in such case is "wrong". If the participant then decides to chose "down" in the next round because her previous guess was "wrong", she would be following the *Win-Stay Lose-Shift* strategy. But, in fact, she is also following *Market Imitation* because she is imitating what market did in the previous round. It is because the two events are mutually exclusive that the performance "correct"-"repeat" actions are also equivalent to *Market Imitation* strategy. S1 Figure E shows an example series illustrating this curious effect where it can directly be seen that at coarse-grained level the two emerging strategies are the same when they are put into practice. However, we must stress that the argument would not work if there were more choices or other possible outcomes in our experimental set up.

Round	1	2	3	4	5	6	7	8	9	10	11	12	13	14	15	16	17	18	19	20	21	22	23	24	25
Market	↑	↑	↓	↓	↑	↓	↓	↓	↓	↑	↑	↓	↑	↑	↓	↑	↓	↑	↑	↑	↓	↓	↓	↑	↓
Player	↓	↑	↑	↓	↑	↑	↑	↑	↑	↓	↑	↓	↓	↑	↓	↓	↓	↑	↑	↓	↑	↑	↑	↓	↑
Result	✗	✓	✗	✓	✓	✗	✗	✗	✗	✗	✓	✓	✗	✓	✓	✗	✓	✓	✓	✗	✗	✗	✗	✗	✗
M.I.		+	+	+		+				+	+		+	+		+			+		+			+	+
W-S L-S		+	+	+		+				+	+		+	+		+			+		+			+	+

S1 Figure E. Coarse-Grained Strategies: "Follow the Strategy" and "Not Follow the Strategy". Here we present an example of a set of actions executed by a random participant along 25 rounds. Market series indicate the price fluctuation of the market. Player indicates the "up"/"down" decision of the participant. Result series display participant's decision coincidences with market (a tick when coincides and a cross when differs). A "+" symbol appears in the row labeled as "MI" when *Market Imitation* strategy has been followed and in the row labeled with "W-S L-S" when *Win-Stays Lose-Switch* strategy has been followed.

Survey Questions

Participants of the experiment were presented a survey about several aspects beyond gender, age and education level. Some of the questions were asked before playing and were obligatory to be answered in order to proceed with the experiment, so in this case the percentages are over the 283 participants. Some other questions could be asked after playing but in that case participants could skip the questions. Thus, the corresponding percentages may not refer over the total number of participants. S1 Figure F shows the answer to the most relevant questions. The rest of the survey questions and answers can be found in the dataset of the experiment.

The last question of S1 Figure F addresses the self-reported degree of importance of intuition and information. S1 Table J displays the conditional probabilities calculated in the main paper segregated by participant's answer in order to cross-check the intuitive nature of the strategies. Unfortunately, any systematic pattern cannot be observed and the majority of probability measures do not deviate from the aggregate case. However, this fact does not disproves main result because it is based on a subjective measure as well as not everybody that participated in the experiment answered to this question.

Answer	p(Up-Up)	p(Down-Down)	p(Sucess-Repeat)	p(Fail-Change)	p(Follow)
Completely intuition	0.726 ± 0.011	0.512 ± 0.014	0.674 ± 0.012	0.590 ± 0.014	0.635 ± 0.009
More intuition	0.696 ± 0.010	0.523 ± 0.012	0.689 ± 0.010	0.544 ± 0.011	0.621 ± 0.008
Same intuition and information	0.718 ± 0.010	0.536 ± 0.013	0.697 ± 0.011	0.575 ± 0.012	0.640 ± 0.008
More information	0.729 ± 0.012	0.586 ± 0.016	0.738 ± 0.012	0.583 ± 0.016	0.671 ± 0.010
Completely Information	0.687 ± 0.021	0.474 ± 0.025	0.597 ± 0.022	0.582 ± 0.027	0.591 ± 0.017
I don't know	0.772 ± 0.018	0.440 ± 0.024	0.688 ± 0.021	0.562 ± 0.023	0.625 ± 0.016
Aggregate	0.717 ± 0.005	0.524 ± 0.006	0.690 ± 0.005	0.570 ± 0.006	0.635 ± 0.004

S1 Table J. Conditional Probabilities Segregated by Self-reported Degree of Importance of Intuition and Information. First column refers to the participant's answer to the last question of S1 Figure F. Second and third columns to the probability to choose "up" after the market has raised and to the probability to choose "down" after a market drop respectively. Fourth and fifth column, account for the probability to repeat a "correct" decision and for the probability to change the previous decision that resulted "wrong". The last column is the probability to follow any of Market Imitation or Win-Stay Lose-Shift strategy as described in the main paper. Last row, "Aggregate" contains the values independently to the answer.

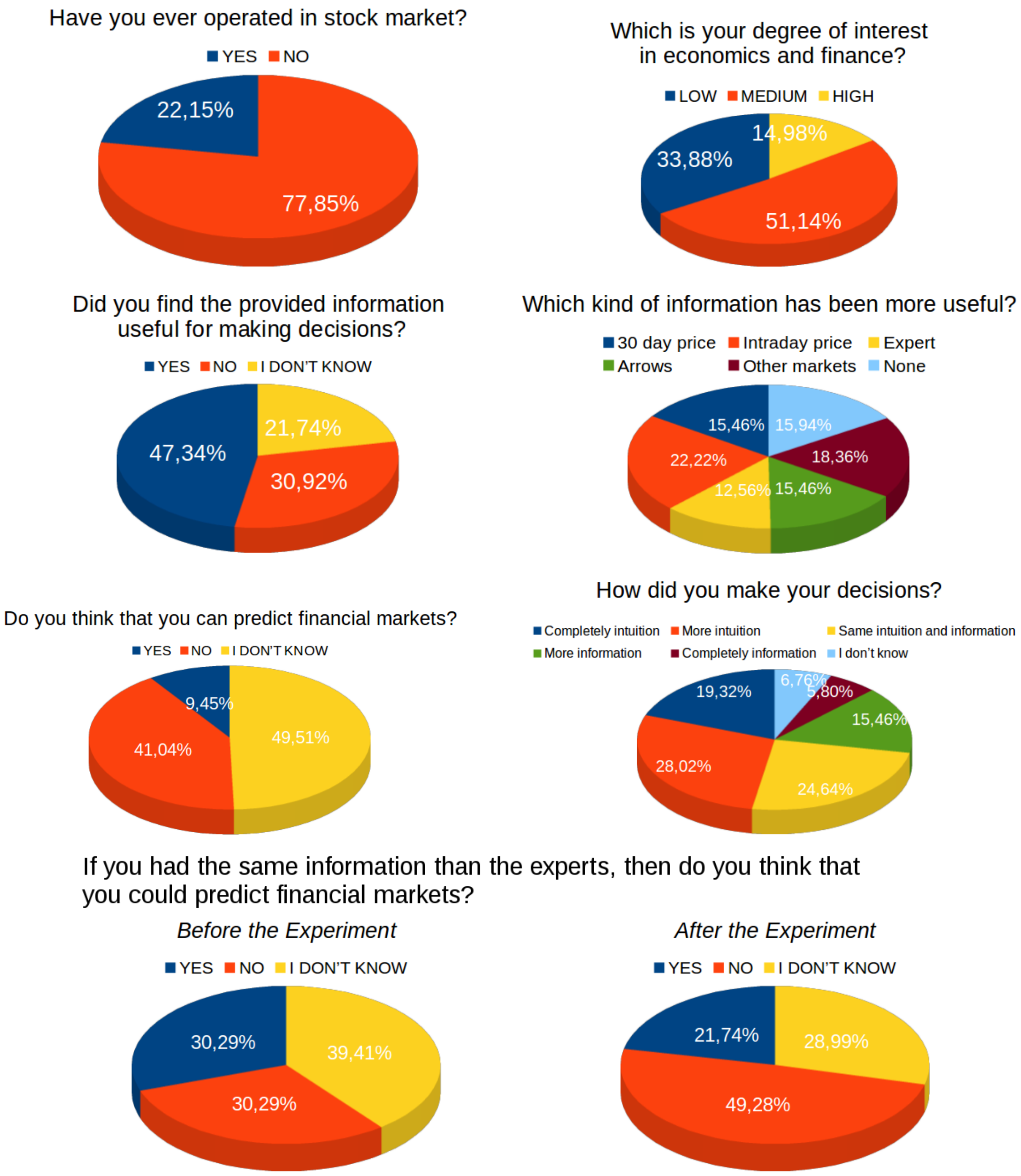

S1 Figure F. Answer percentage to some questions in the survey. The two questions on the top were asked before playing the game and could not be skipped. The rest of the questions were asked after playing and the participant could voluntarily exit the survey.

Mutual Information

Mutual information is a measure of the reduction in uncertainty of a certain random variable due to the knowledge of another random variable and can be understood as a measure of the dependence between them.[1] Thus, in order to know if participants are influenced by the market's previous action ("up" or "down") and the participant's previous own outcome ("right" or "wrong") we have computed the mutual information value for several cases but paying special attention the emerging strategies presented in the main paper. Mutual information is defined as

$$I(X,Y) = \sum_{x,y} p(x,y) \log \frac{p(x,y)}{p(x)p(y)} = \sum_{x,y} p(y|x)p(x) \log \frac{p(y|x)}{p(y)} \quad (4)$$

where X and Y are the two random variables. It is defined positive and takes values between 0 and 1, meaning that both random variables are completely independent or that they are perfectly correlated respectively. Mutual information values are given in bits units since we have used the logarithm with base two.

In order to study *Market Imitation* strategy we compute the mutual information involving the market's (M_{n-1}) previous action, at round $n-1$, and the nth decision D (see S1 Figure G). The resulting value obtained for the mutual information is:

$$I(M_{n-1}, D_n) = 0.045 \pm 0.010 \text{ bits}, \quad (5)$$

which corresponds a great deviation of 4.5 SD units from 0.

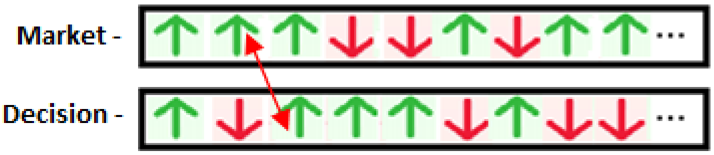

S1 Figure G. Strategy based on market's movement: the Market Imitation strategy. We show a possible market's sequence and a possible decision's path. Green arrows pointing "up" mean that the market rises (first row) and that the participant has made an "up" guess (second row). Red arrows pointing "down" mean that the market falls (first row) or it that the participant has made a "down" guess (second row). The inclined red arrow shows the displacement relation studied by measuring the mutual information.

Regarding the strategy *Win-Stay Lose-Shift*), we study the influence of the previous result R_{n-1} on the next decision D_n expressed in terms of "repeat" or "change" (see S1 Figure H). The mutual information value obtained in this case is:

$$I(R_{n-1}, D_n) = 0.050 \pm 0.010 \text{ bits}, \quad (6)$$

which is also a big difference of 5 SD from 0.

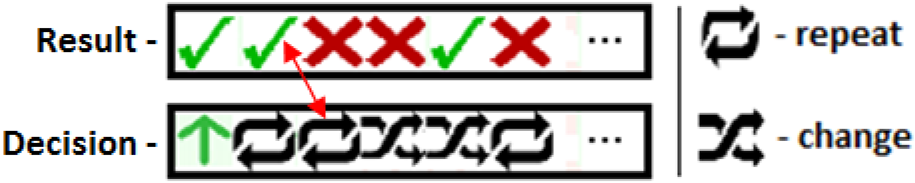

S1 Figure H. Strategy based on previous outcome: the Win-Stay Lose-Shift strategy. We show a possible result sequence where actions are "stay" (or "repeat") and "shift" (or "change") previous decision. The inclined red arrow shows the displacement relation being studied. It has to be noted that the first decision has been discarded as it has no market previous action to be compared with.

Those results prove that the decision process is not independent of the market evolution nor the success process and therefore *Market Imitation* and *Win-Stay Lose-Shift* emerging strategies are relevant in the decision-making process. Furthermore, we have also computed the mutual information of the market with itself, $I(M_{n-1}, M_n) = 0.003 \pm 0.010$ bits, and also the mutual information of the participant's own action, $I(D_{n-1}, D_n) = 0.005 \pm 0.010$ bits to see what information encode these processes alone. In both cases, we can observe that these values are not significantly different from zero with. Thus, nor the market nor the decision alone encode information about next action.

It has finally to be noted that we have computed these values taking into account the previous market's action and participant's outcome. Thus, we have computed the conditional probabilities contemplating these two events one round before, say at rounds $n-1$ and n, the decision event (for example, one of the probabilities computed is $p(\uparrow_n \mid \uparrow_{M,n-1})$). Additionally, we have checked that the mutual information algorithm has been correctly implemented with two different codes. The first one generates randomly a market sequence and a decision path so that when computing the mutual information the expected value is 0 as the sequences are independent. The second code generates a market sequence randomly and a decision path following exactly what the market has done in the previous round. In this case the expected value is 1 as they are perfectly correlated. Since in both cases these values have been obtained with a discrepancy of just the $0,01\%$, it can be considered that the mutual information algorithm used throughout the study is correct.

For what it concerns to participant's memory, we could wonder if what happened two rounds before still influences the next decision. In order to quantify this, we have recomputed the mutual information values for both strategies but this time considering the influence of round $n-2$ on round n. For the *Market Imitation* strategy we have obtained a mutual information value of 0.001 ± 0.009 bits and for the *Win-Stay Lose-Shift* strategy, 0.002 ± 0.010 bits. These two values are, again, not differentiable from zero, saying that these strategies are mainly one-step memory processes. See Section 'Ìnformation Loss and Two-step Markov Chains" for further details.

Conditional Mutual Information: the interdependence between both pairs of sequences

Here we study the dependence of the decision's path on the market's action (related to the *Market Imitation* strategy) and the outcome sequence (related to the *Win-Stay Lose-Shift* strategy) separately as if the market and the outcome affected independently the participant. Nevertheless, it could be possible that these two processes were affected by each other and that when considering both influences more information is encoded. In order to see if taking both processes into account increases the information or at least to better know which is the dominant strategy, we compute the *conditional* mutual information

$$I(X,Y|Z) = \sum_{x,y,z} p(x,y,z) \log_2 \frac{p(x,y,z)p(z)}{p(x,z)p(y,z)}. \tag{7}$$

Thus, we study the influence of the outcome on the market-decision (*Market Imitation*) comparison $I(M_{n-1}, D-n|R_{n-1})$ and also the influence of the market's action on the outcome-decision (*Win-Stay Lose-Shift*) comparison $I(R_{n-1}, D_n|M_{n-1})$. As before, in these two values both the market and the outcome belong to round '*n-1*' while the action belongs to the *nth* round. The *conditional* mutual information obtained are respectively

$$I(M_{n-1}, D_n|R_{n-1}) = 0.05 \pm 0.04 \text{ bits}, \tag{8}$$

and

$$I(R_{n-1}, D_n|M_{n-1}) = 0.07 \pm 0.04 \text{ bits}. \tag{9}$$

Both values are positive which means that the market and the outcome sequence add non-redundant information. However, we shall also point that the market's behavior adds more information, so that *Market Imitation* strategy seems to be more relevant in our experiment.

Conditional Probabilities and Mutual Information: A brief discussion

To better understand the $I(M_{n-1}, D_n)$ and $I(R_{n-1}, D_n)$ values, the conditional probabilities for the decision being "up" or "down" knowing the market's previous direction or the participant's previous outcome and the marginal probabilities for the two versions of the decisions' path have been computed. It can be seen that these probabilities are statistically relevant in all cases compared to the marginal probabilities, as shown in S1 Table K. This can make us think that the mutual information values obtained are too low, but the fact is that the mutual information is a sum of terms weighted by its respective joint probabilities and so it can be interpreted as an average in which no special cases stand out. Let us highlight again the behavioral bias related to these probabilities which is already discussed in the main paper: the $p(D_n^u|M_{n-1}^u)$ is much higher than $p(D_n^d|M_{n-1}^d)$. Thus, it can be said that there is a general tendency to follow the market when it is rising that has made participant's think the market will keep on this upward trend for at least one more round.

Information Loss and Two-step Markov Chains

Up to now we have observed that the knowledge of the events of the previous round reduces uncertainty about the participant's next decision, specially when considering particular cases. Such encoding of the information suggests that participants have

Conditional probabilities	Marginal probabilities
Decision and market's events	
$p(D_n^u \vert M_{n-1}^u) = 0.714 \pm 0.005$	
$p(D_n^d \vert M_{n-1}^u) = 0.286 \pm 0.005$	$p(D_n^u) = 0.607 \pm 0.004$
$p(D_n^u \vert M_{n-1}^d) = 0.469 \pm 0.006$	$p(D_n^d) = 0.393 \pm 0.004$
$p(D_n^d \vert M_{n-1}^d) = 0.531 \pm 0.006$	
Decision and outcome's events	
$p(D_n^{rp} \vert R_{n-1}^r) = 0.682 \pm 0.005$	
$p(D_n^{ch} \vert R_{n-1}^r) = 0.318 \pm 0.005$	$p(D_n^{rp}) = 0.561 \pm 0.004$
$p(D_n^{rp} \vert R_{n-1}^w) = 0.421 \pm 0.006$	$p(D_n^{ch}) = 0.439 \pm 0.004$
$p(D_n^{ch} \vert R_{n-1}^w) = 0.579 \pm 0.006$	

S1 Table K. Comparison between conditional probabilities and marginal probabilities. In reference to the *Market Imitation* strategy, we summarize the conditional probabilities of the decision (D) being "up" (u) or "down" (d) for the nth round having the market (M) raised (u) or fallen (d) in the round $n-1$. While in relation to the *Win-Stay Lose-Shift* strategy, we also present the conditional probabilities to "repeat" (or "stay", 'rp') same action or to "change" (or "shift", 'ch') decision D having the previous outcome (R) been "right" ('r') or "wrong" ('w'). Finally, the marginal probabilities for the "up" or "down" guesses and for "change" or "repeat" the previous decision are also presented.

Decision conditioned to... two previous market's movement	two previous outcomes and one previous action	
$p(D_n^u \vert M_{n-1}^u, M_{n-2}^u) = 0.709 \pm 0.006$	$\mathbf{p(D_n^{rp} \vert R_{n-1}^r, D_{n-1}^{rp}, R_{n-2}^r) = 0.715 \pm 0.008}$	$p(D_n^{rp} \vert R_{n-1}^r, D_{n-1}^{rp}, R_{n-2}^w) = 0.670 \pm 0.012$
$p(D_n^d \vert M_{n-1}^u, M_{n-2}^u) = 0.291 \pm 0.006$	$\mathbf{p(D_n^{ch} \vert R_{n-1}^r, D_{n-1}^{rp}, R_{n-2}^r) = 0.285 \pm 0.008}$	$p(D_n^{ch} \vert R_{n-1}^r, D_{n-1}^{rp}, R_{n-2}^w) = 0.330 \pm 0.012$
$\mathbf{p(D_n^u \vert M_{n-1}^d, M_{n-2}^u) = 0.493 \pm 0.008}$	$p(D_n^{rp} \vert R_{n-1}^w, D_{n-1}^{rp}, R_{n-2}^r) = 0.412 \pm 0.010$	$\mathbf{p(D_n^{rp} \vert R_{n-1}^w, D_{n-1}^{rp}, R_{n-2}^w) = 0.550 \pm 0.013}$
$\mathbf{p(D_n^d \vert M_{n-1}^d, M_{n-2}^u) = 0.507 \pm 0.008}$	$p(D_n^{ch} \vert R_{n-1}^w, D_{n-1}^{rp}, R_{n-2}^r) = 0.588 \pm 0.010$	$\mathbf{p(D_n^{ch} \vert R_{n-1}^w, D_{n-1}^{rp}, R_{n-2}^w) = 0.450 \pm 0.013}$
$p(D_n^u \vert M_{n-1}^u, M_{n-2}^d) = 0.716 \pm 0.007$	$\mathbf{p(D_n^{rp} \vert R_{n-1}^r, D_{n-1}^{ch}, R_{n-2}^r) = 0.526 \pm 0.014}$	$\mathbf{p(D_n^{rp} \vert R_{n-1}^r, D_{n-1}^{ch}, R_{n-2}^w) = 0.715 \pm 0.010}$
$p(D_n^d \vert M_{n-1}^u, M_{n-2}^d) = 0.284 \pm 0.007$	$\mathbf{p(D_n^{ch} \vert R_{n-1}^r, D_{n-1}^{ch}, R_{n-2}^r) = 0.474 \pm 0.014}$	$\mathbf{p(D_n^{ch} \vert R_{n-1}^r, D_{n-1}^{ch}, R_{n-2}^w) = 0.285 \pm 0.010}$
$\mathbf{p(D_n^u \vert M_{n-1}^d, M_{n-2}^d) = 0.447 \pm 0.008}$	$p(D_n^{rp} \vert R_{n-1}^w, D_{n-1}^{ch}, R_{n-2}^r) = 0.408 \pm 0.014$	$\mathbf{p(D_n^{rp} \vert R_{n-1}^w, D_{n-1}^{ch}, R_{n-2}^w) = 0.350 \pm 0.011}$
$\mathbf{p(D_n^d \vert M_{n-1}^d, M_{n-2}^d) = 0.553 \pm 0.008}$	$p(D_n^{ch} \vert R_{n-1}^w, D_{n-1}^{ch}, R_{n-2}^r) = 0.592 \pm 0.014$	$\mathbf{p(D_n^{ch} \vert R_{n-1}^w, D_{n-1}^{ch}, R_{n-2}^w) = 0.650 \pm 0.011}$

S1 Table L. Conditional probabilities of the decision considering the last two rounds. We show the conditional probabilities of the decision (D) depending on the market's (M) or the outcome's (R) events in the last two steps. The superindex 'rp' mean "repeat" action, the superindex 'ch' means "change" action, the superindex 'r' means that the guess was "right" and 'w' that it was "wrong". The values in bold are those statistically relevant (and different) compared to the values shown in S1 Table K.

memory of the last round and that this influences their strategy. But now it can be asked whether this memory is wider or not, i.e., if, for instance, events that have taken place at round $n-2$ can tell something about the *nth* decision. In order to study how previous rounds are actually relevant on the decision, the mutual information with the market's action and the participant's outcome at round $n-2$ have also been computed and we thus obtain

$$I(M_{n-2}, D_n) = 0.001 \pm 0.009 \text{ bits}, \tag{10}$$

and

$$I(R_{n-2}, D_n) = 0.002 \pm 0.010 \text{ bits}. \tag{11}$$

We observe that these values are not different from 0 and, thus, in general no information about the next decision is encoded in the two rounds before. Therefore, it can be said that only the previous round influences the participant's decision, so its memory holds for only one round. Nevertheless, as said before, the mutual information is a thermalized value and in some special cases memory could be wider. For that reason, we next look at possible behavioral biases by considering the conditional probabilities $p(D_n|M_{n-1}, M_{n-2})$ and $p(D_n|R_{n-1}, D_{n-1}, R_{n-2})$. Results are shown S1 Table L. We can observe that, when comparing the probabilities that represent the same event in the nth and $n-1$th rounds, in some cases (values in bold) these conditional probabilities are statistically relevant from the ones shown in S1 Table K while in some other cases they are not. That is to say that, for example, we have compared $p(D_n^u|M_{n-1}^d, M_{n-2}^u)$ with $p(D_n^u|M_{n-1}^d)$ and $p(D_n^{rp}|R_{n-1}^r, D_{n-1}^{ch}, R_{n-2}^w)$ with $p(D_n^{rp}|R_{n-1}^r)$. In the case of the outcome-decision comparison (*Win-Stay Lose-Shift* strategy), we note that the decision of the $n-2$ step has also

Both strategies conditional probabilities	
$p(D_n^u \mid M_{n-1}^u, R_{n-1}^r)$	0.729 ± 0.005
$p(D_n^d \mid M_{n-1}^u, R_{n-1}^r)$	0.271 ± 0.005
$p(D_n^u \mid M_{n-1}^u, R_{n-1}^w)$	0.679 ± 0.007
$p(D_n^d \mid M_{n-1}^u, R_{n-1}^w)$	0.320 ± 0.007
$p(D_n^u \mid M_{n-1}^d, R_{n-1}^r)$	0.415 ± 0.008
$p(D_n^d \mid M_{n-1}^d, R_{n-1}^r)$	0.585 ± 0.008
$p(D_n^u \mid M_{n-1}^d, R_{n-1}^w)$	0.520 ± 0.007
$p(D_n^d \mid M_{n-1}^d, R_{n-1}^w)$	0.479 ± 0.007

S1 Table M. Double conditional probability in terms of the outcome and the market direction. We show the conditional probabilities by considering the decision (D) jointly depending on the market's (M) or the outcome's (R) events in the previous step. The superindex 'u' means "up" action, the superindex 'd' means "down" action, the superindex 'r' means that the guess was "right" and 'w' that it was "wrong".

been considered since we are interested in knowing how the participant adapts at each step its strategy and thus if, for example, it tends to "repeat" its decision when it has been successful. It is in this case remarkable that the probability of repeating the decision when the outcome has been "correct", increases when this situation takes place two consecutive times compared to when it only happens once. In a similar way, the probability of changing the decision when the participant has failed to predict also increases when these events take place twice. Therefore, it seems like there is a tendency to adapt decisions depending on their success. On the other hand, the probability of following the market's rising trend of the two previous rounds is not differentiable from that of considering only the previous round. Contrarily, the probability of following the market's tendency when it has fallen twice increases with respect to considering only the previous step. These facts can be understood as there is some distrust to believe that the upward trend will keep for more rounds, although this probability is still high, while if the tendency is downwards the reliance on this trend increases.

Reproducing the Mr.Banks Experiment at the CAPS event

As described in Methods Section of the main paper, Mr.Banks experiment was reproduced during the course of the annual event of the European project "Collective Awareness Platforms for Sustainability and Social Innovation" (CAPS2015) that took place in 7 and 8 July 2015 hosted in Brussels (Belgium). A space of 20 square meters at the venue entrance was prepared to carry out the experiment Mr.Banks(2). In total, 42 attendants of the conference participated in the experiment, which implied the sum of 2,372 decisions. We present here the main results from this second experiment. First, the behavioral bias of the participants observed in the DAU experiment is present again in this one. Indeed, the probability to choose "up" in CAPS 2015 is $p(\uparrow) = 0.605 \pm 0.013$ in agreement with the main experiment DAU 2013 where $p(\uparrow) = 0.606 \pm 0.004$. Second, success ratio also matches the results as values fall inside error range since the empirical probability in DAU 2013 was 0.536 ± 0.004 while in CAPS 2015 is 0.527 ± 0.014. However, the most important result is that the probabilities that conform both strategies are very similar, as shown in S1 Figure I. The difference of such probabilities between DAU 2013 and CAPS 2015 is not significant except for the case of changing ("shift" event) the decision after a "wrong" guess in the *Win-Stay Lose-Shift* strategy. In this case, empirical probabilities differ in 0.044 which corresponds to 2.78 SD units. Nonetheless, in all cases and without exception, the probabilities follow a similar pattern and they are significantly higher than the reference values. We can confirm that the explored emerging strategies we observe in the DAU data are also present in a very different context such as the CAPS 2015 conference.

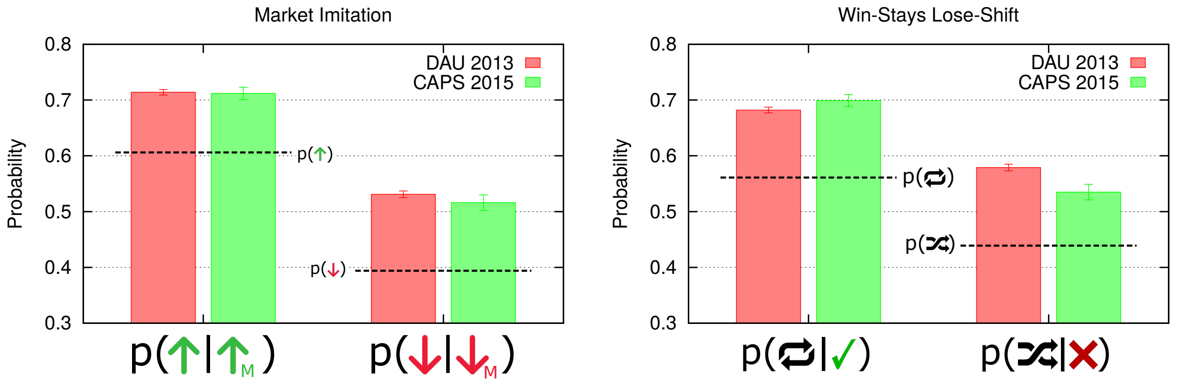

S1 Figure I. Comparing the emerging Strategies in DAU 2013 and CAPS 2015 experiments. (Left) Conditional probabilities of *Market Imitation* strategy are plotted. The reference value of the marginal probability of guessing "up" or "down" are shown with black dashed lines. (Right) Conditional probabilities of *Win-Stay Lose-Shift* strategy are plotted. The reference value of the unconditioned probability of the "stay" and "shift" actions are shown with black dashed lines. In all cases, error bars indicate the Standard Deviation (SD).

Mr. Banks tutorial

In S1 Figs. J, K, L, M and N, we show the different screens that were used as the tutorial for the Mr. Banks participants. In these screens we present what types of information were available for the participants, and how this information was consulted.

References

1. Thomas M. Cover & Joy A. Thomas, *Elements of information theory*, 2nd ed. (John Wiley and sons, New York, 1991).

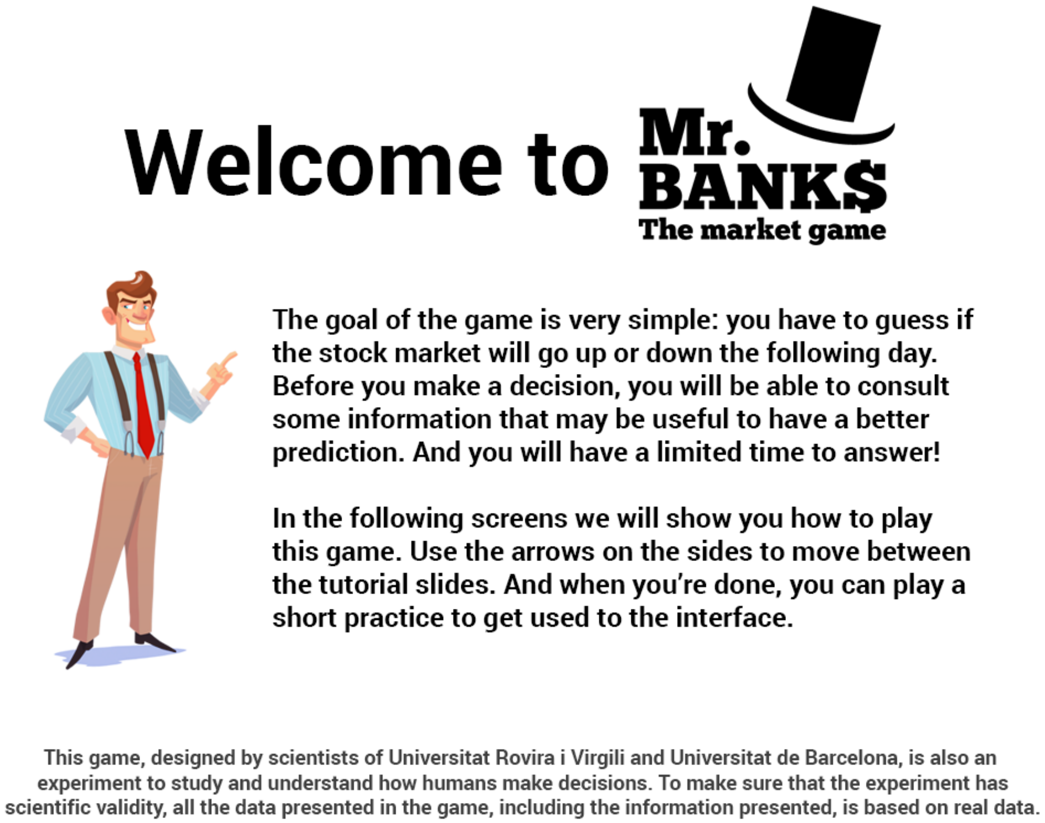

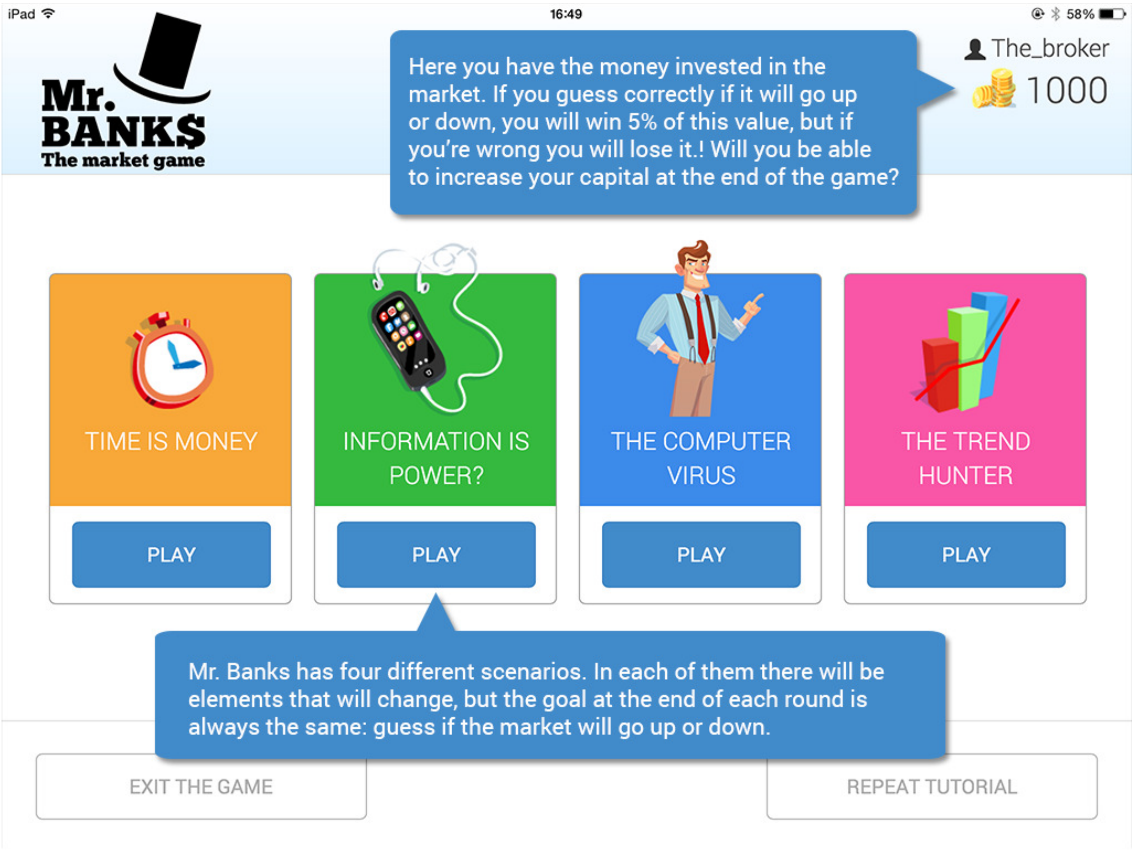

S1 Figure J. **Screens 1 and 2 of the Mr. Banks tutorial.**

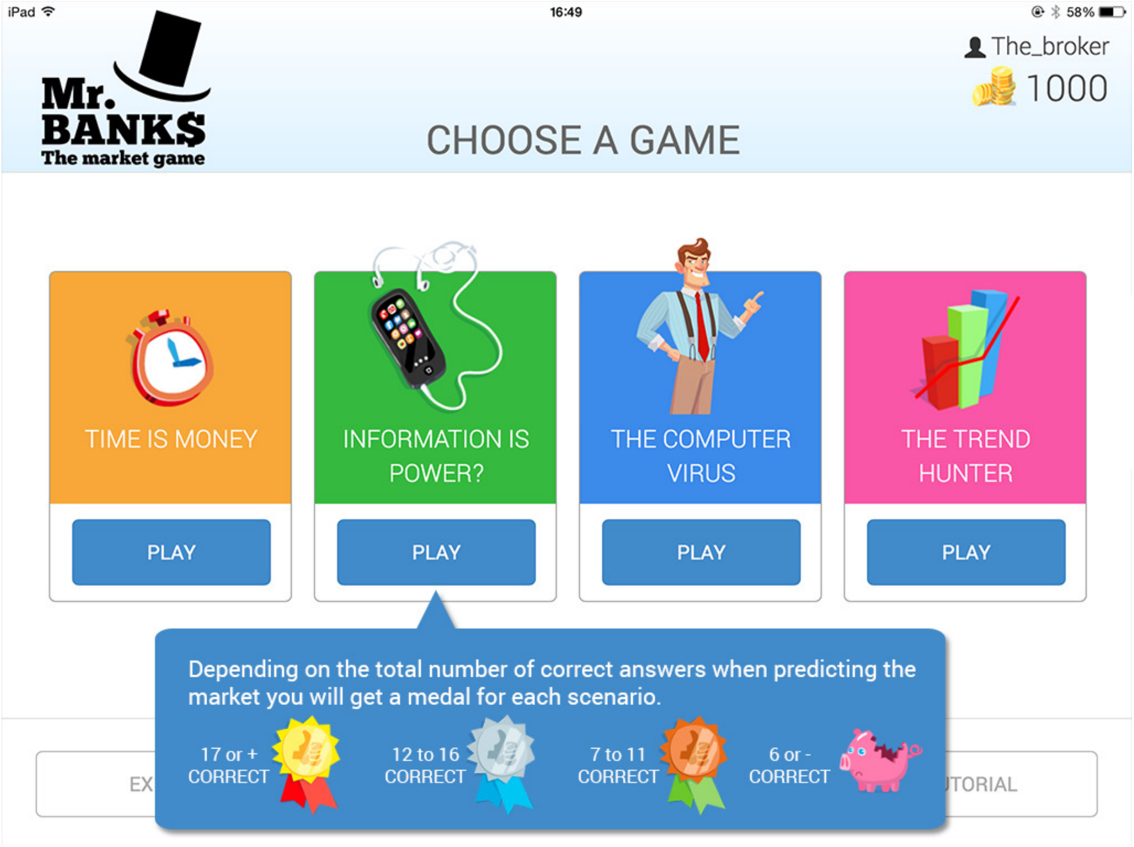

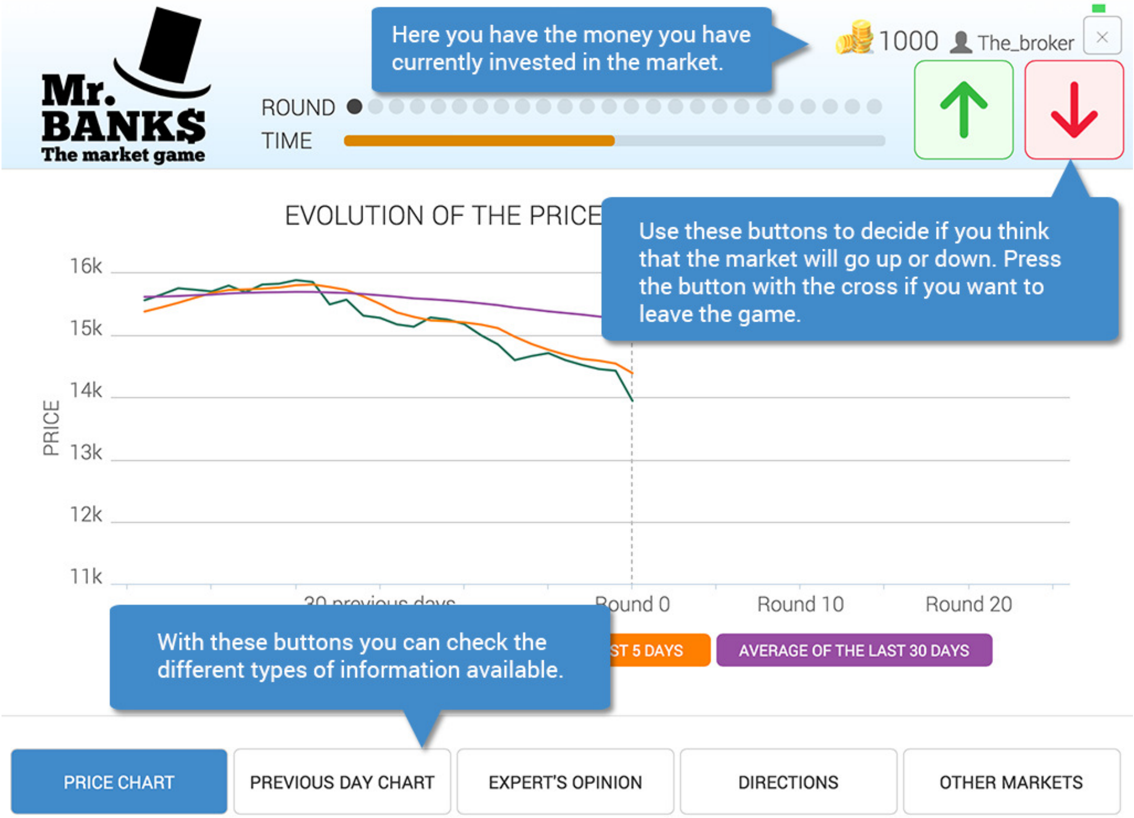

S1 Figure K. Screens 3 and 4 of the Mr. Banks tutorial.

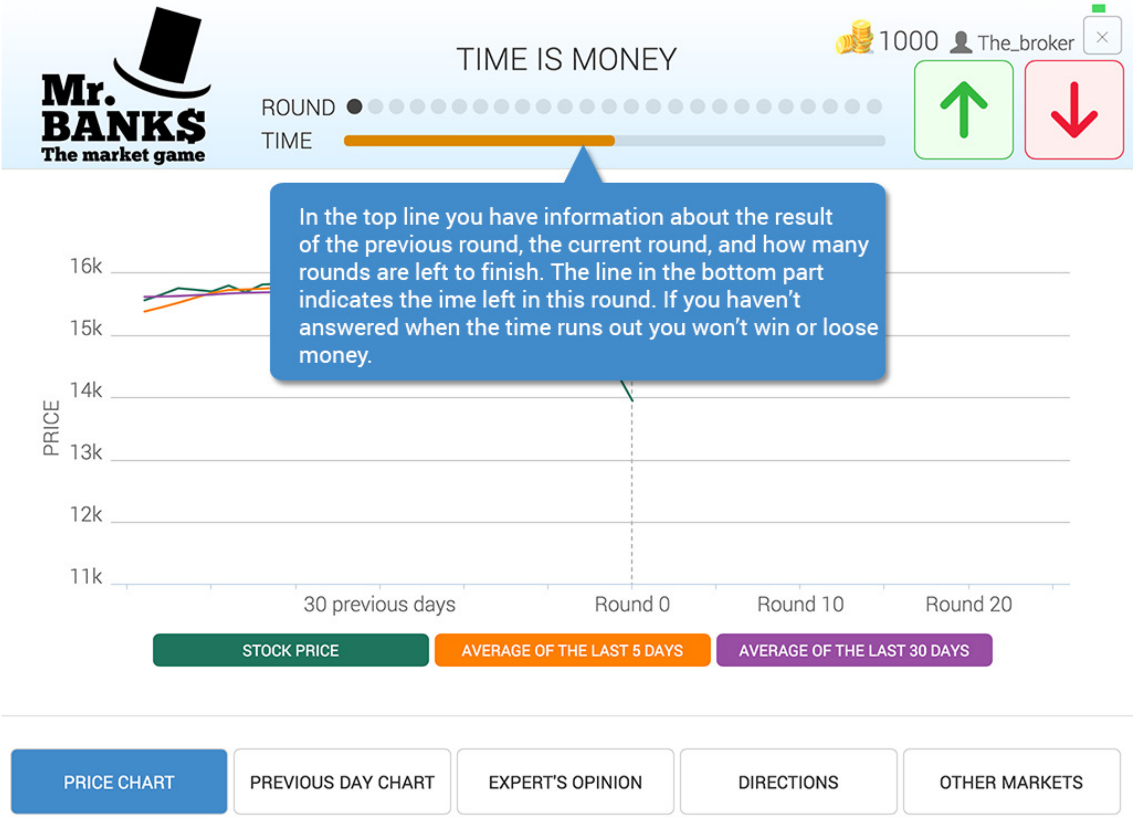

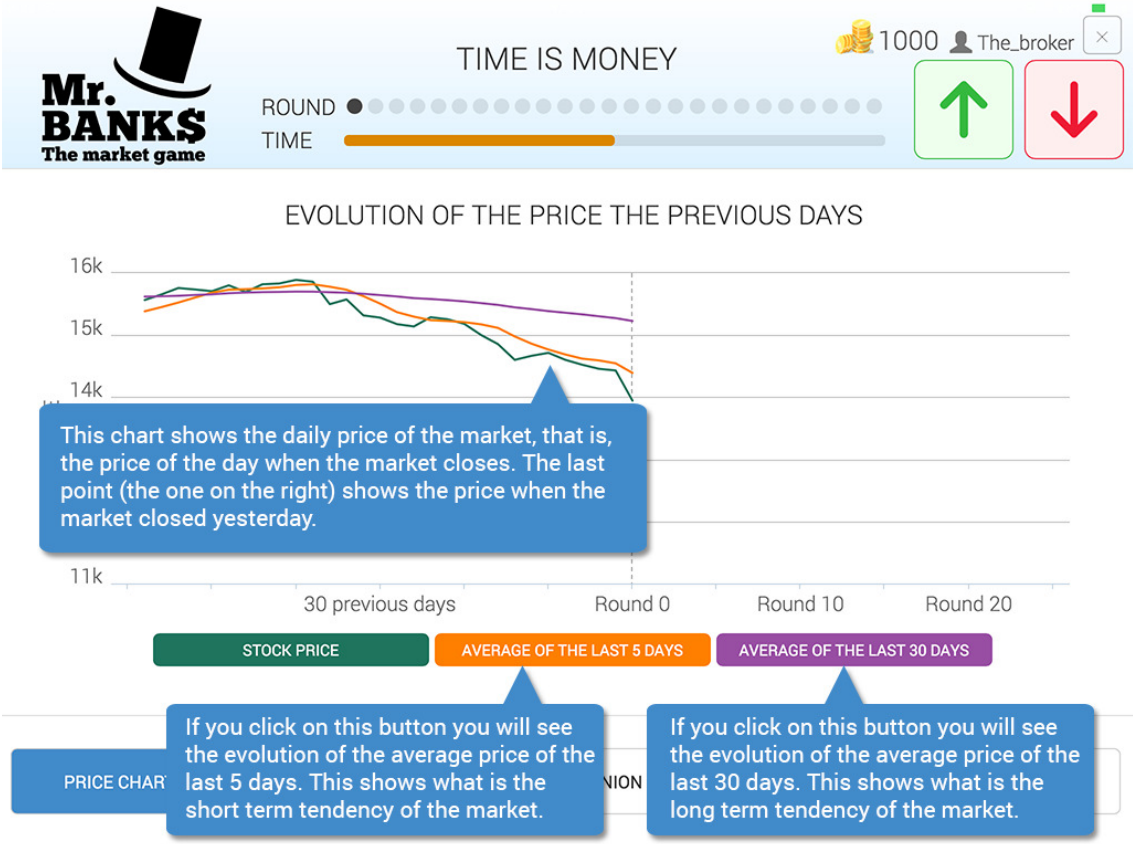

S1 Figure L. Screens 5 and 6 of the Mr. Banks tutorial.

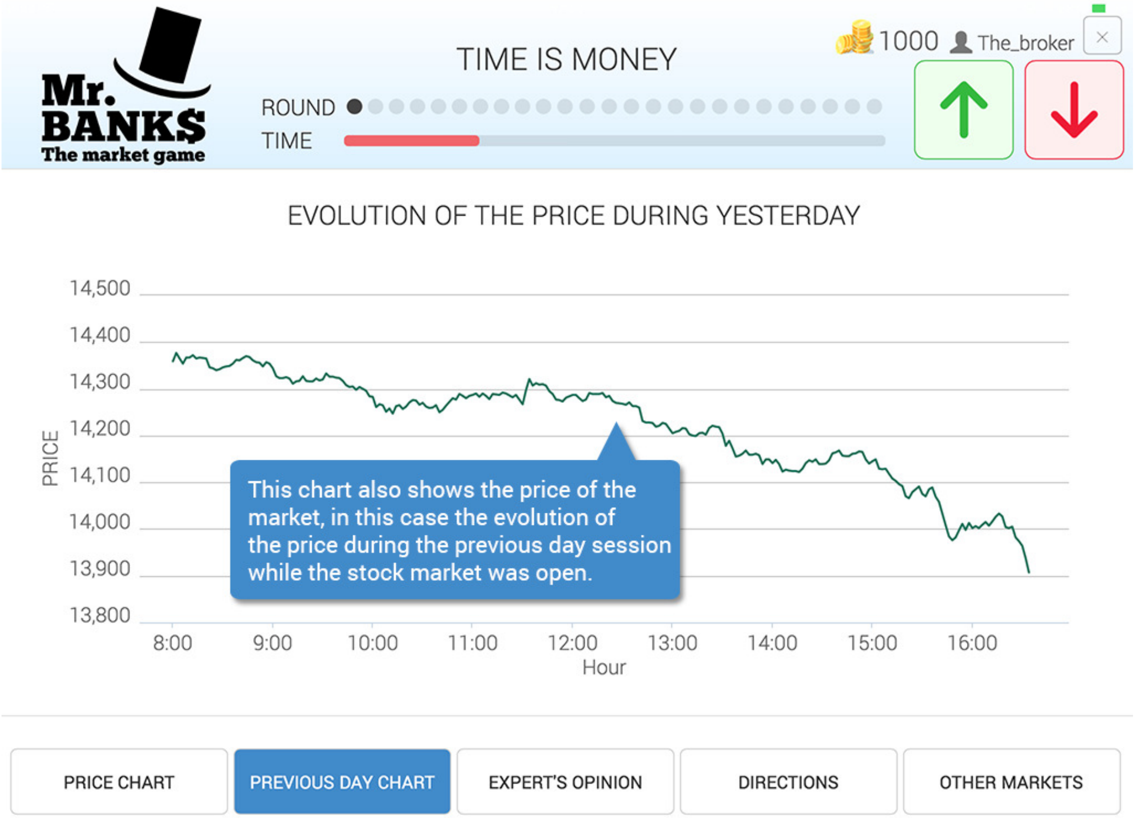

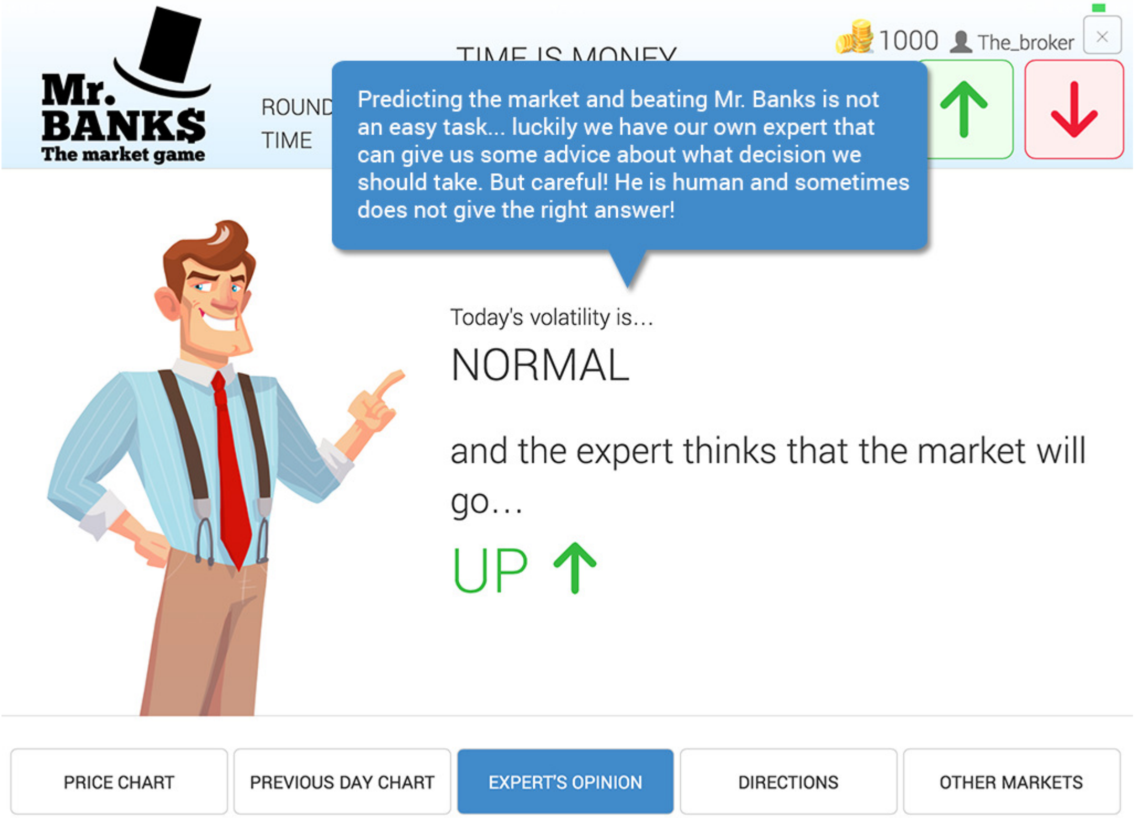

S1 Figure M. Screens 7 and 8 of the Mr. Banks tutorial.

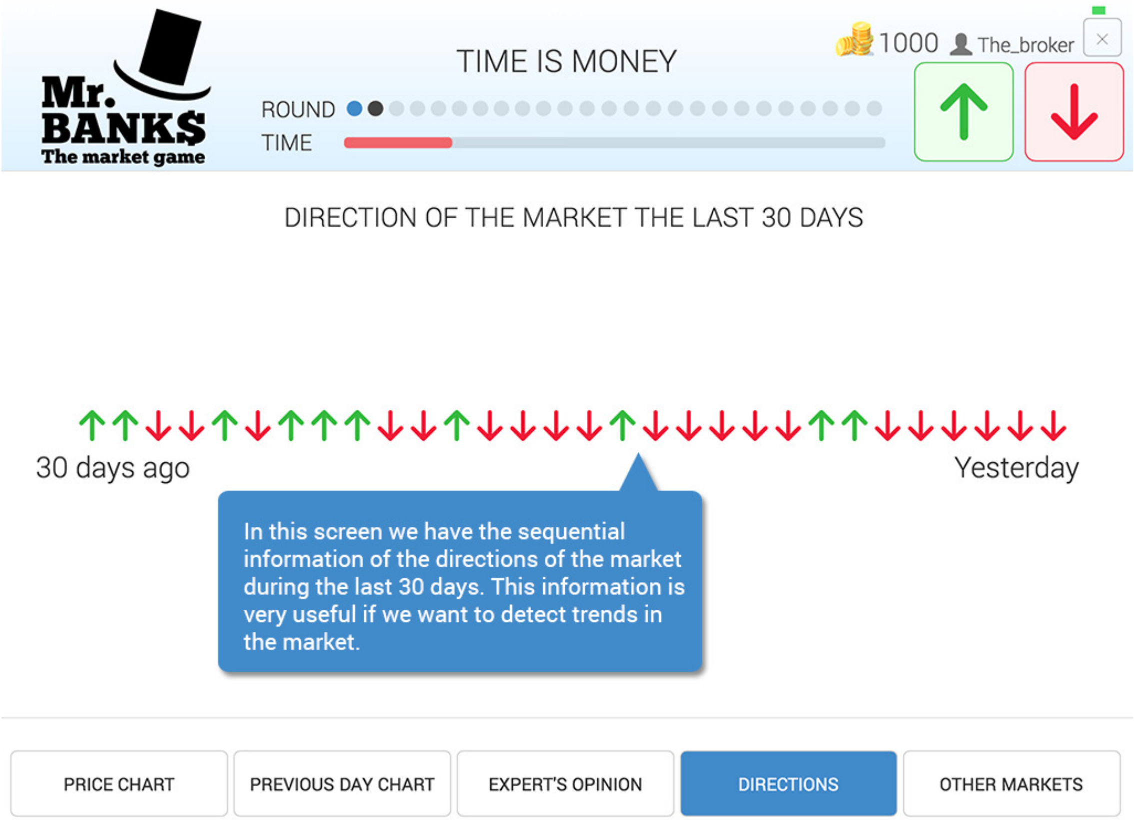

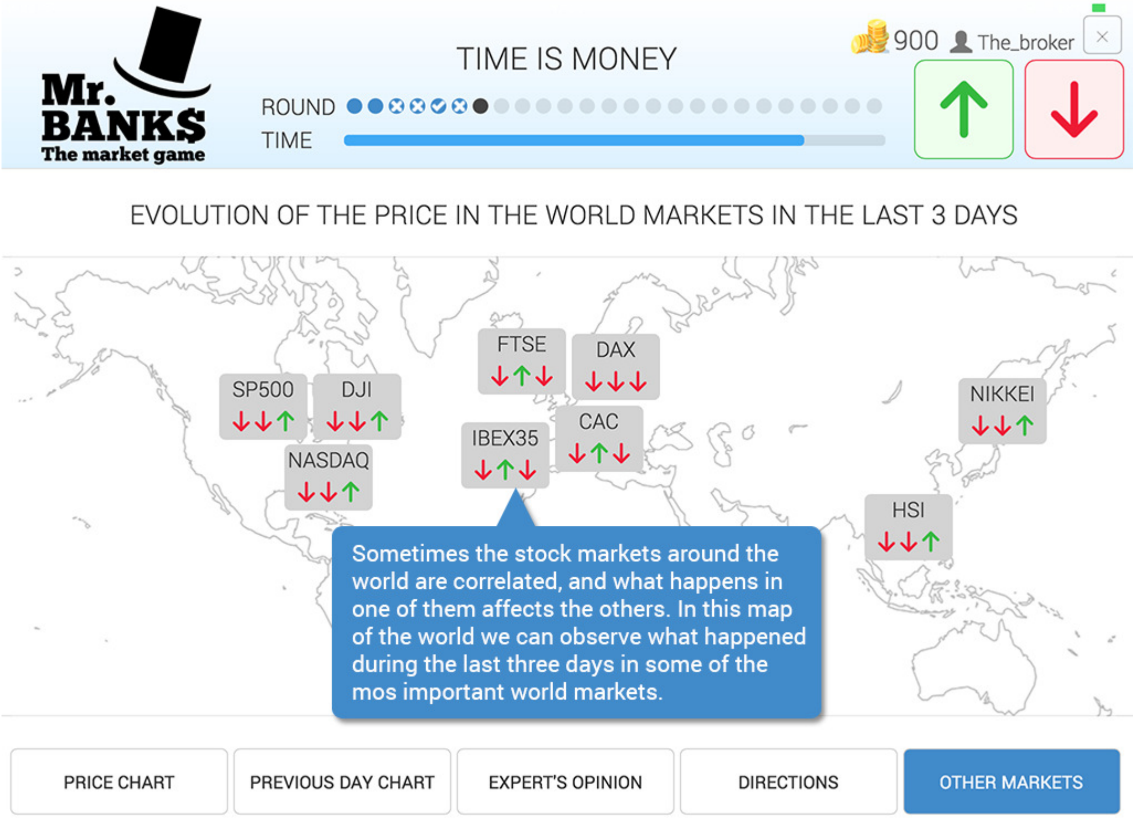

S1 Figure N. Screens 9 and 10 of the Mr. Banks tutorial.
